# Vascular impulse technology versus elevation for reducing the swelling of upper and lower extremity joint fractures

**DOI:** 10.1038/s41598-022-27231-6

**Published:** 2023-01-12

**Authors:** Jan S. El Barbari, Marc Schnetzke, Moritz B. Bergmann, Lukas Baumann, Sven Y. Vetter, Benedict Swartman, Paul A. Grützner, Jochen Franke

**Affiliations:** 1 Department for Traumatology and Orthopaedics, BG Klinik Ludwigshafen, Ludwig-Guttmann-Str. 13, 67071 Ludwigshafen, Germany; 2 German Joint Center, ATOS Clinic Heidelberg, Bismarckstr. 9, 69115 Heidelberg, Germany; 3 Institute of Medical Biometry, University of Heidelberg, Im Neuenheimer Feld 130, 69120 Heidelberg, Germany; 4 Medical Faculty, University of Heidelberg, Im Neuenheimer Feld 346, 69120 Heidelberg, Germany

**Keywords:** Randomized controlled trials, Outcomes research

## Abstract

Soft-tissue conditioning due to posttraumatic oedema after complicated joint fractures is a central therapeutic aspect both pre- and postoperatively. On average, 6–10 days pass until the patient is suitable for surgery. This study compares the decongestant effect of vascular impulse technology (VIT) with that of conventional elevation. In this monocentric RCT, 68 patients with joint fractures of the upper (n = 36) and lower (n = 32) extremity were included and randomized after consent in a 1:1 ratio. Variables were evaluated for all fractures together and additionally subdivided into upper or lower extremity for better clinical comparability. Primary endpoint was the time in days from hospital admission to operability. Secondary endpoints were total length of stay, oedema reduction, pain intensity, complications, and revisions. The time from admission until operability was reduced by 1.4 (95% CI − 0.4; 3.1) days in the mITT analysis (p = 0.120) and was statistically significant with 1.7 (95% CI 0.1; 3.3) days in the as-treated sensitivity analysis (p_AT_ = 0.038). Significantly less pain and a faster oedema reduction were found in the intervention group. Due to rare occurrences, nothing can be concluded regarding complications and revisions. Administration of VIT therapy did not lead to a significant reduction in time until operability in the whole population but was superior to elevation for soft-tissue conditioning and pain reduction. However, there was a significant reduction by 2.5 days (95% CI 0.7; 4.3) in the subgroup of lower extremity fractures. VIT therapy therefore seems to be a helpful tool in the treatment of posttraumatic oedema after complex joint fractures of the lower and upper extremity, especially in tibial head and lower leg fractures.

## Introduction

Soft-tissue swelling in the setting of complex joint fractures is a critical factor in clinical management^1–8^. The exerted mechanical forces due to the trauma cause direct damage to the surrounding soft tissues and vessels, leading to hematoma formation and primary swelling^9–12^. As a result of the developing inflammatory response, there is an increase in capillary permeability via the release of coagulative factors and inflammatory mediators, and thus a secondary swelling due to an accumulation of fluid in the interstitial space^13^. This gives rise to a clinical soft-tissue swelling, which leads to a rising tissue pressure and a reduced microcirculation due to venous stasis, and thus to tissue hypoxia and local acidosis^13^.

This impairs wound and bone healing, and the accumulation of metabolites causes increased pain^14–16^. If either the trauma is so severe that a vicious circle is formed from this cycle, or if an attempt is made to perform osteosynthesis in this setting, this can lead to the development of the maximal degree of soft-tissue trauma: compartment syndrome^12,17,18^. Because of this, joint fractures require rigorous soft-tissue conditioning until adequate decongestion has been achieved. Only then is surgical intervention possible without inadequately compromising the soft tissues. The traditional methods consisting of rest, elevation, cooling, and compression are mainly used as decongestant measures. These are effective and rheologically proven, especially for elevation^19,20^.

However, all these measures have a purely passive effect, by lowering the hydrostatic pressure, and are therefore both lengthy and only effective as long as they are consistently applied. In the 1970s, a venous plexus was demonstrated for both venous return from the foot and hand. Their physiology was proven in further studies using phlebography and duplex sonography^21–24^. In either case, the effect unfolds through a combination of passive and active moieties, which gave rise to the name venous hand or foot pump^25–27^. For the foot pump, the passive effect consists of weight bearing during the stance phase and the stretching of the plantar surface during roll-off, draining the contained blood volume proximally. In the hand, the superficial veins, both palmar and dorsal, pass over prominent bony structures and are compressed onto them during fist clenching^24,28^.

Synergistically with this passive moiety, the blood from the deep intermuscular veins in both regions is actively transported centrally by muscle pumps^27^. Since their development, there have been numerous studies on the use of intermittent pneumatic compression (IPC) devices, the Vascular Impulse Technology therapy (VIT therapy) being one form of these. This system consists of an air compressor connected to a pad placed into the palm of the hand or the sole of the foot with a bladder that is inflated every 20 s in less than 1 s to exert a pressure of 130 mmHg on the venous plexus of the foot and 80 mmHg on the hand, respectively. This causes a pulsatile blood flow and measurements have revealed that the effect reaches the right atrium^21–24^. These sufficiently demonstrate their effect on thromboprophylaxis in general and especially in the context of arthroplasty^29–33^. Less research has been done on their benefit in perioperative trauma care. Sufficient data for clinical benefit exist for foot and ankle fractures as most recently published by Schnetzke et al.^34–39^.

Only a few studies exist for other anatomical locations, further proximal on the lower leg and tibial plateau, as well as for joint fractures of the upper extremity. Their validity is further limited by methodological weaknesses, small case numbers or only a limited indication^40–44^. They all demonstrated significantly faster oedema resolution, an increased range of motion, and less pain^10,45–47^. In some cases, a volume reduction of 70–90% of the trauma-induced soft-tissue swelling could be achieved^41,43^. However, some studies, though having distinct methodologic flaws, showed no significant benefit^48,49^. Thus, evidence of the benefit of VIT therapy in this setting can be called ambiguous or sparse at best.

Therefore, and due to the lack of evidence for upper extremity fractures, the benefit of VIT therapy in complex fractures of the lower leg and the upper extremity was investigated with regard to decongestion, perioperative length of stay, pain intensity and medication, and complication and revision rates. It was hypothesized that VIT therapy would reduce the time in days until operability was achieved.

## Results

The intervention and control groups were comparable in demographic variables (age, type of injury, gender, academic, comorbidities, smoker. See Tables 1 and 2). Upon evaluation of the primary endpoint in the mITT population of the entire study group, the intervention group showed a mean reduction of 1.4 (CI − 0.4; 3.1) days in the length of stay until operability, with 6.7 ± 2.9 days compared to 8.0 ± 3.8 days in the control group (p = 0.120, see Table 3). In the subsequent sensitivity analysis in the AT population, the difference was significant, with a reduction of 1.7 (CI 0.1; 3.3) days in the intervention group. (p_AT_ = 0.038).

**Table 1:** Demographic variables.

	Intervention (n_VIT_ = 35)	Control (n_K_ = 31)
Age, mean (SD)	57.0 (11.5)	57.8 (12.8)
**Injury, n (%)**		
Tibial plateau	14 (40.0%)	12 (38.7%)
Lower leg	3 (8.6%)	1 (3.2%)
Humerus		
Proximal	7 (20.0%)	6 (19.4%)
Distal	3 (8.6%)	2 (6.5%)
Elbow	3 (8.6%)	4 (12.9%)
Distal radius	5 (14.3%)	6 (19.4%)
**Sex, n (%)**		
Male	8 (22.9%)	11 (35.5%)
Female	27 (77.1%)	20 (64.%)
**Academic, n (%)**		
Yes	8 (22.9%)	5 (16.1%)
No	27 (77.1%)	26 (83.9%)
**Comorbidities, n (%)**		
Yes	18 (58.1%)	17 (50.0%)
No	13 (41.9%)	17 (50.0%)
**Smoker, n (%)**		
Yes	9 (25.7%)	12 (38.7%)
No	26 (74.3%)	19 (61.3%)
**Side, n (%)**		
Right	8 (22.9%)	11 (35.5%)
Left	27 (77.1%)	20 (64.5%)

**Table 2:** Comorbidities, complications, and revisions in detail.

	Intervention (n_VIT_ = 35)	Control (n_K_ = 31)
**Comorbidities, n**		
High blood pressure	10	13
Hypothyroidism	3	8
Diabetes	4	2
Previous cardiovascular Injury	4	6
Others (single mentions)	11	13
Complication (revision)	Swelling Compartment Syndrome (Fasciotomy)	Swelling Pulmonary embolism

**Table 3:** Primary and secondary endpoints.

	n_VIT_/n_K_	Intervention	Control	p value
**Primary endpoint**				
Time from admission until operability, mean (SD) (days)				
mITT	32/29	6.7 (2.9)	8.0 (3.8)	0.120^a^
AT	24/37	6.3 (2.4)	8.0 (3.8)	**0.038**^**a**^
Lower extremity (LE)				
mITT	17/13	7.8 (2.2)	10.3 (2.5)	**0.009**^**a**^
AT	13/17	7.6 (1.7)	9.9 (2.8)	**0.011**^**a**^
Upper extremity (UE)				
mITT	15/16	5.3 (3.0)	6.2 (3.8)	0.490^a^
AT	11/20	4.7 (2.2)	6.4 (3.8)	0.145^a^
**Secondary endpoints**				
Length of stay, mean (SD) (day)				
All	35/31	14.9 (6.8)	15.7 (6.9)	0.629^a^
LE	17/13	18.9 (6.1)	20.4 (5.7)	0.512^a^
UE	18/18	11 (5.1)	12.3 (5.6)	0.478^a^
Decongestion, mean (SD) (%/day)				
Total	23/24	10.3 (8.7)	6.3 (9.8)	0.050^a^
Preop	23/24	10.0 (10.5)	5.6 (10.4)	0.163^a^
Postop	19/22	10.7 (6.0)	7.1 (9.2)	0.148^a^
Pain-free, n (%) (VAS)				
Preop	23/23	9 (40%)	3 (13%)	**0.044**^**b**^
Postop	19/23	12 (63%)	1 (4%)	** < 0.001**^**b**^
Complications^c^, n (%)	35/31	2 (5.7%)	2 (6.5%)	–
Revisions^c^, n (%)	35/31	1 (2.9%)	0 (0%)	–

Significant values are in bold.

^a^Welch’s *t-test* for unequal variances, ^b^Mann–Whitney U Test, ^c^percentages are calculated according to the cases that occurred in the mITT population.

**Figure 1:**
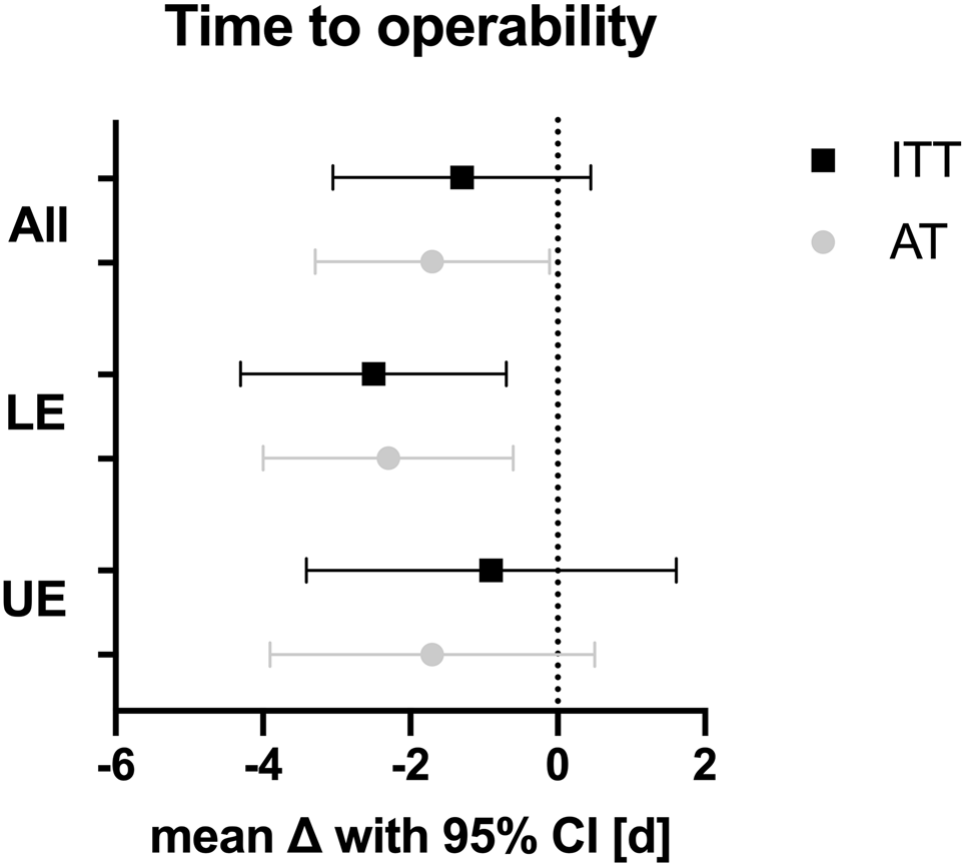
Comparison of the mean reduction achieved by VIT in the delay to surgery with a graphed 95% confidence interval. *ITT* intention-to-treat population, *AT* as-treated population, *LE* lower extremity, *UE* upper extremity.

Analysis of the subgroups showed a significant reduction in favor of the intervention group in the lower extremity fractures with a difference of 2.5 (CI 0.7; 4.3) days (p = 0.009: see Table 3), but no significant difference could be detected with these numbers in the upper extremity with 0.9 (CI − 1.6; 3.4) days in favor of the intervention group (p = 0.490). The mean difference between the intervention and control group is shown in Fig. 1.

Taking the entire inpatient stay into account, there was also a reduction of 0.8 (CI − 2.6; 4.2) days in the intervention group, with 1.4 (CI − 3.0; 5.9) in the lower and 1.3 (CI − 2.3; 4.9) in the upper extremity respectively, but these were not significant (p_total_ = 0.629; p_LE_ = 0.512; p_UE_ = 0.478).

**Figure 2:**
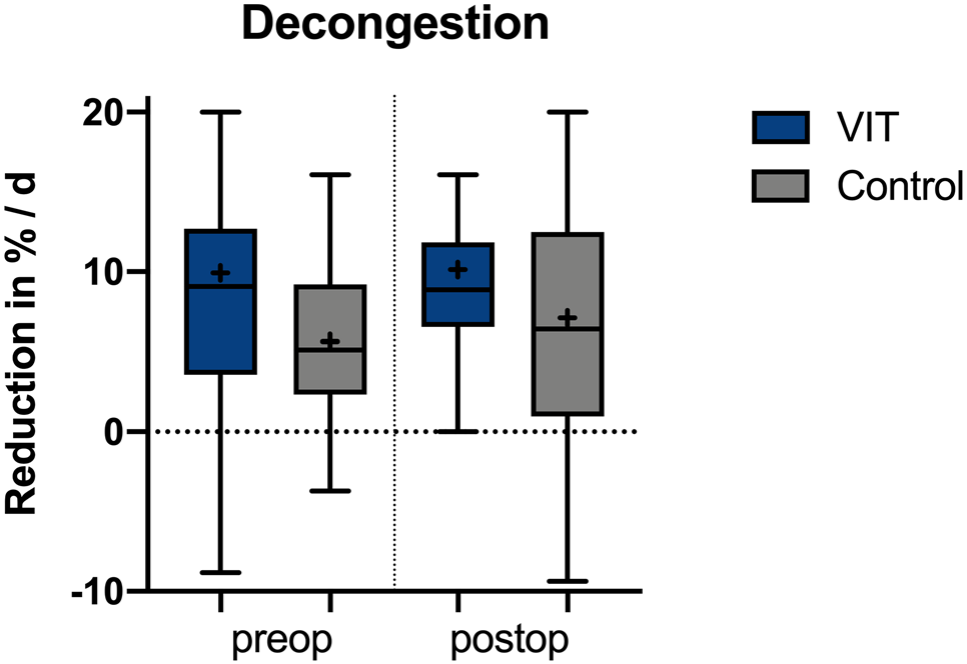
Boxplots of the reduction of posttraumatic oedema pre- and postoperatively of both groups, measured in percentage per day.

Preoperatively a nearly two-fold increase in decongestion could be seen in the intervention group with a reduction of posttraumatic oedema of around 10.0 ± 10.5% per day instead of 5.6 ± 10.4% per day, which was not statistically significant (p = 0.163). Postoperatively there was an increase of about 50% in favor of the intervention group (10.7 ± 6.0%/d instead of 7.1 ± 9.2%/d, p = 0.148; see Fig. 2).

The overall mean increase in decongestion (pre- and postoperatively) by 3.9 (CI 0.001; 7.9; p = 0.050) was significant, with 10.3 ± 8.7% per day in the intervention group compared to 6.4 ± 9.8% per day in the control. In relation to mean use of the VIT therapy, this can be calculated as a reduction in swelling of 4.41 ± 15.41% per hour of VIT therapy.

A significant difference could be found in patients being pain-free preoperatively with a ratio of 40% (9/23) in the intervention group compared to 12% in the control group (3/26; p = 0.044) and upon discharge with 63% (12/19) compared to 4% (1/23; p < 0.001).

Only a slight numerical difference regarding opioids needed preoperatively could be seen in the intervention group with 26% (6/23) of the patients requiring pain medication according to step 3 of the WHO scheme, compared to 32% (8/25) in the control group (p = 0.756). Upon discharge, this difference rose to 5.6% (1/18) vs. 18.2% (4/22; p = 0.356).

Due to the low numbers, no statistical analysis could be performed concerning the complication and revision rates. There were two complications in each of the two groups resembling a rate of 11.8% in the intervention group and 15.4% in the control group. However, there was only one revision (fasciotomy) in one of the intervention group patients (5.9%, 1/17) due to noncompliance to the therapy protocol. The patient concerned used the VIT for only 1 h/day and regularly left the surgical ward to smoke cigarettes.

The multiple linear regression analysis (duration ~ group + demographic characteristics; Table 4) showed no difference in the primary endpoint regarding “age”, “gender”, “education level”, “comorbidities”, “smoker”, and “affected side”.

**Table 4:** Regression analyses.

Linear regression analyses Model: time of hospitalization to readiness for surgery ~ Group (reference control group) + characteristic			
Model variables	Estimator^a^	95% CI	p value
Group	− 1.39	− 3.12; 0.35	0.114^b^
Age	− 0.01	− 0.08; 0.06	0.764^b^
Group	− 1.44	− 3.28; 0.41	0.125^b^
Gender (ref. men)	0.17	− 1.73; 2.07	0.859^b^
Group	− 1.38	− 3.11; 0.36	0.119^b^
Education level	− 0.08	− 2.26; 2.10	0.942^b^
Group	− 1.33	− 3.09; 0.42	0.134^b^
Comorbidities	− 0.82	− 0.94; 2.58	0.356^b^
Group	− 1.49	− 3.24; 0.27	0.095^b^
Smoker	− 0.67	− 2.53; 1.20	0.479^b^
Group	− 1.41	− 3.15; 0.33	0.110^b^
Affected side (ref. right)	0.37	− 1.54; 2.27	0.702^b^

^a^Interpretation of the estimator: for categorial variables, the estimator indicates the change in time from hospitalization to operability in the variable indicated compared with the reference category. Therefore in the Time ~ Group + Age model, for example, the time from hospitalization to operability in the VIT group is reduced by 1.39 days (because of a negative sign) compared with the control group. For continuous variables, the estimator describes the change in the time from hospitalization until operability with an increase in the continuous variables by 1. ^b^t-test.

## Discussion

This study aimed to explore if administering VIT therapy as a form of intermittent pneumatic compression would improve soft-tissue conditioning and could consequently reduce the time until operability would be achieved. Following the discovery of venous return from the foot and the hand by means of both an active and passive venous pump, studies were carried out that tried to stimulate these mechanisms and improve and accelerate soft-tissue conditioning as well as thromboprophylaxis, especially during the posttraumatic immobilization period. The focus was primarily on ankle fractures, since the soft-tissue envelope is rather thin and perfusion critical here, leading to high complication rates due to posttraumatic oedema. However, there have only been limited studies on other lower extremity fractures and even less on the upper extremity.

For tibial plateau and shaft fractures, only one published study with solely the abstract but not the full text in English could be found. This described a significantly faster reduction in swelling preoperatively in 68 cases of tibial and fibular fractures^50^. Seven studies could be identified in the last 40 years which focused on fractures of the upper extremity^40–44,48–50^. The effects of intermittent pneumatic compression were examined in lymphedema after mastectomy^40^, after operative treatment of Dupuytren’s contracture^44^, Colles’ fracture^42^, for 48 h perioperatively in distal radius fractures^41^, and only in one case after surgical treatment of distal radius fractures in the form of a prospective randomized trial^43^. In summary, a significantly improved postoperative decongestion, an improved venous return during therapy, and relieved pain with increased range of motion were found^40–44^.

Nonetheless, in two of these studies, posttraumatic oedema was not examined^40,44^. The work of Svensson et al. showed an effect in postoperative treatment, yet no therapy was given preoperatively^42^. In the study of Ramesh et al. the therapy was administered perioperatively, though no control group was included and the studied period covered just 48 h^41^. The first prospective randomized trial was conducted by Mader et al., but there was no control for elevation, nor a preoperative treatment. Additionally, it only included patients who were initially treated by an external fixator and presented preoperatively with an increased girth due to swelling of at least 3 cm^43^.

Only two recent studies could be identified^48,49^. Both could not demonstrate a significant or at least relevant benefit of the VIT therapy. In the first study, however, therapy was only applied after 4 weeks of postoperative immobilization in distal radius fractures^48^ and in the second study, a pressure of 20 mmHg was applied to the venous hand plexus, which does not resemble a physiologic stimulation^49^.

Thus, this study is the first to examine the effect of VIT therapy in the perioperative management of different complex fractures of the lower and upper extremity compared to elevation using a physiologic stimulation of the venous pump in the foot and hand in a prospective, controlled, and randomized setting.

This physiologic stimulation can be achieved by a fast pulsatile compression of the venous plexus as would result from weight bearing in case of the foot or clenching of the hand. This impulse induces shear stress, which in turn releases NO-reducing tissue hypoxia and improved blood flow^21–24^. Moreover, therapy was administered throughout the entire perioperative period right after hospital admission and so exactly that period in which an impaired micro- and macro-circulation might cause complications^13–16^. In previous studies, a continuous pressure was used or therapy only applied in a confined and not directly posttraumatic period.

It could be shown that the delay to surgery could be reduced by 1–2.5 days. The difference was higher in the lower extremity subgroup for two reasons: swelling was more severe and so the venous pump was more effective and, in general, the delay to surgery in the upper extremity was rather short with a mean of 5–6 days. Decongestion was significantly improved by approximately 50% from which a mean reduction in swelling of around 4.5% per hour use of the VIT therapy can be calculated. Hence, the beneficial effect of intermittent pneumatic compressions on posttraumatic oedema that could only be anticipated from former studies in these fractures could now be further substantiated and proven.

As described in a previous study, the improved microcirculation with fewer metabolites accumulating in the tissue is supposed to be the trigger of the lesser pain reported in the intervention group with three times as many patients reported being pain-free peroperatively and upon discharge^13^. No effect was found in regard to complication rates and revisions needed.

### Limitations

A major limitation of the study in the case of upper extremity fractures is that only a fraction of the targeted group size of 20 patients per fracture could be achieved. Thus, only a limited power could be achieved in these groups with regard to the statistical analysis. On the one hand, this was because patients with such fractures were often too old or comorbid. On the other hand, and more importantly, this was because these patients were in most cases not admitted as inpatients for soft-tissue conditioning but were immobilized in a plaster cast and treated as outpatients. However, even in these cases, significant effects could be detected. The second limitation is the lack of blinding and hence the risk of performance bias. However, blinding was not feasible for either the observer or the participant due to the obvious intervention. This limitation was countered by selecting independent observers to assess the operability. A correlation analysis carried out in a preliminary study (κ = 0.816) revealed almost complete agreement in their assessments. Another limitation is that the pure circumference measurement performed to objectify the swelling was rounded to 5 mm increments. However, a volumetric analysis was not possible when patients were immobilized in a cast or external fixator and a more precise measurement was not possible due to the duration and size of the study and the fact that it was carried out in the context of normal inpatient daily routine.

## Conclusion

Although no significant overall reduction in the delay until operability could be shown, there was a significant reduction by 2.5 days in complex fractures of the tibial head and lower leg. Significant improvement in decongestion by more than 50% and in pain-free patients preoperatively and upon discharge could be seen in both upper and lower extremity fractures. Due to the low incidence, no statement regarding complications and revisions could be made.

In short, Vascular Impulse Technology is an effective additional tool for soft-tissue conditioning, especially in fractures of the lower leg with a more profound swelling.

## Methods

This prospective, randomized, controlled, monocentric study included a total of 68 patients with upper (n = 36) and lower limb (n = 32) fractures from 2016 to 2019. These were enrolled and randomized upon admission in permuted blocks of equal length to the intervention (n = 35) and control (n = 33) groups in a 1:1 ratio using the “Randoulette” program from the University of Munich (https://wwwapp.ibe.med.uni-muenchen.de/randoulette/index.jsp). Blinding was not possible due to the acoustically and visually obvious intervention. The study was pre-registered in the German Clinical Trials Registry (DRKS00010510; 18/07/2016) and approved by the Ethics Committee of the Rhineland-Palatinate Medical Association (837.155.16/10474) as the supervising committee for medical studies at the BG Trauma Center Ludwigshafen. The study protocol was published before the study was started^51^.

After informed consent, patients aged between 18 and 80 years with complex fractures of the lower leg shaft (n = 4), tibial plateau (n = 28), proximal (n = 13) and distal (n = 5) humerus, with ligamentous and bony elbow dislocations (n = 7) and distal radius fractures (n = 11) who could not be definitively treated by ORIF on the day of admission were included. Since patients with upper limb injuries were mostly managed as outpatients preoperatively, patients were also included from inpatient admission to surgery after decongestion (n = 5) due to the small number of cases.

Exclusion criteria were met if patients were younger than 18 or older than 80 years, had not signed an informed consent, had concomitant injuries to the contralateral side, or had open fractures with pre-existing soft-tissue complications, such as infections, stress blisters, necrosis or compartment syndrome. Patients with decompensated heart failure, thrombosis or pulmonary artery embolism, or acute phlebitis were not included. Participants in other studies, pregnant women, prison inmates, and drug or alcohol abusers were not included either.

**Figure 3:**
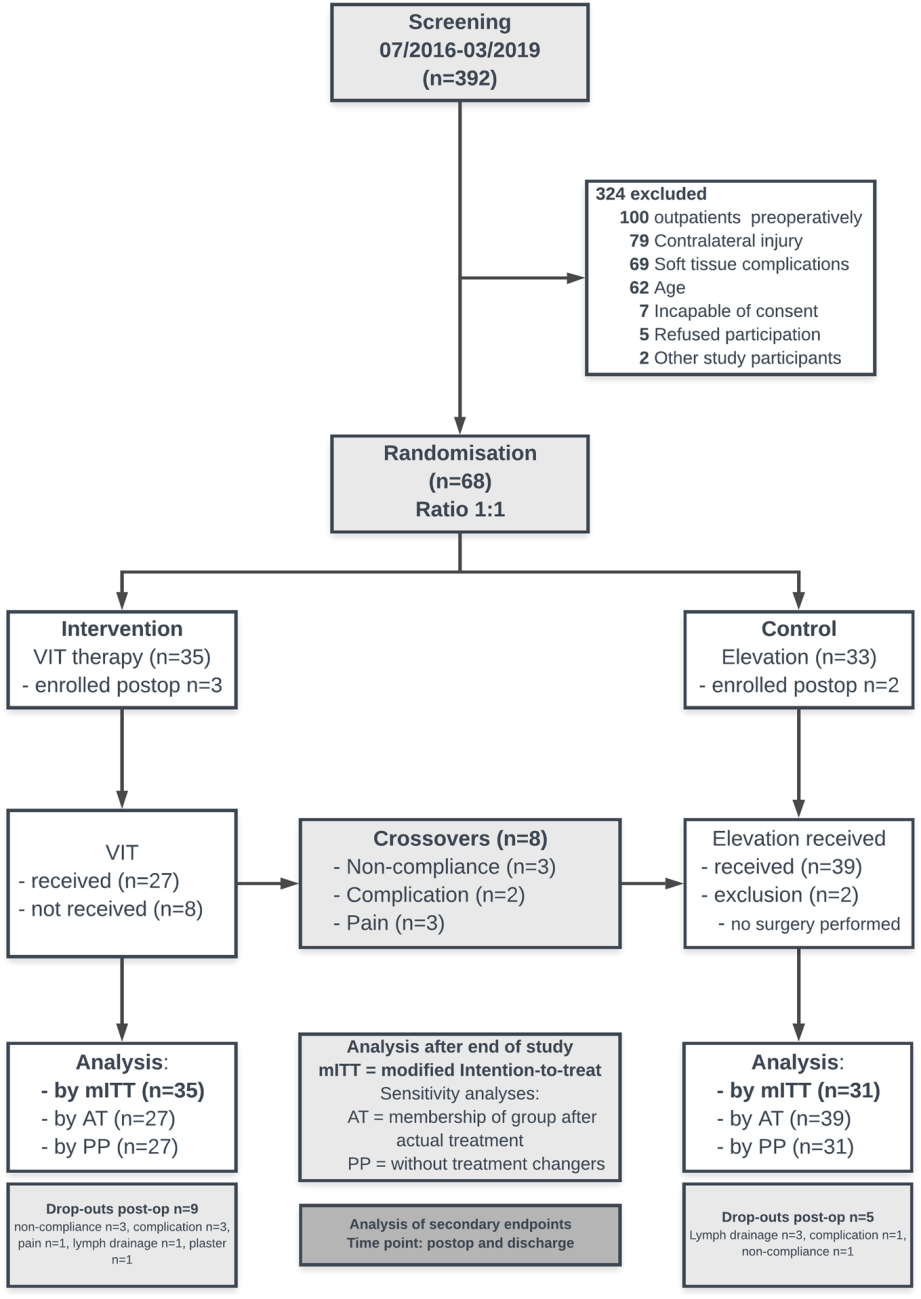
Flowchart of the VIT study.

Figure 3 shows a flow chart of the study with data collection and drop-outs. After allocation to one of the groups, patients in the control group were only placed with the injured extremity in elevation; no further measures to reduce swelling, such as cooling or compression were carried out. Patients in the intervention group received a 15-min instruction in the VADOplex system (Fa. OPED GmbH, Oberlaindern, Germany), which they were required to use preoperatively without interruption if possible and postoperatively for at least 6–8 h daily. This air compressor inflates and deflates a pad with an air bladder attached to the sole of the foot or palm of the hand via Velcro straps to a pressure of 130 or 80 mmHg, respectively, within one second at intervals of 20 s. Stable fractures were immobilized in a splint or cast. Unstable fractures were reduced and immobilized with an external fixator until definitive surgical treatment.

The time from hospital admission to operability in days was defined as the study’s primary endpoint. Operability was evaluated by an independent observer (one of two senior physicians in trauma surgery who were not involved in the study) during the daily ward round. As practiced in preliminary studies, this was evaluated as soon as the normal skin wrinkling became apparent again^52,53^. In a preliminary study, almost complete agreement (κ = 0.816) was found between the two observers regarding the assessment of operability^39^.

The secondary endpoints (1) length of stay (days), (2) pain intensity (VAS), (3) pain medication (according to WHO step scheme), (4) soft-tissue swelling (cm) to calculate oedema reduction (%/d), (5) complications (n), and (6) revisions (n) were collected during a daily study visit. Swelling of the soft tissues was measured in cm circumference and, derived from this, reduction in swelling was evaluated as a percentage per day. For the evaluation of soft-tissue swelling in lower extremity fractures, the standardized measurement points at the level of the medial knee joint space and at the smallest tibial circumference were used. For proximal and distal humerus fractures, these points were 15 cm proximal and at the elbow level, and for elbow and distal radius fractures at the elbow level and 10 cm distally. These were the sites of greatest swelling and the measurement points closest to the fracture, thus the most clinically relevant. The mean percentage swelling at these points was assessed and further evaluated. Girth measurements were used since volumetric measurement was not suitable. This was because the unstable fractures included were reduced using an external fixator and measurements were obtained during daily work on the surgical ward.

In a previously conducted study, sample size calculation led to a group size of 17 patients per group to detect a clinically relevant and statistically significant difference of 2 ± 2 days induced by VIT therapy^39,51^. Although this number had been calculated for fractures of the ankle joint, it was also applied for the total number of patients in this study due to insufficient data for other anatomic regions.

### Statistics

All data were described with suitable measurements of central tendency and dispersion. All confidence intervals (CIs) presented are 95% confidence intervals.

Data were analyzed for all fractures as a whole, as well as separately for the upper and lower extremities. All statistical tests were performed with a two-sided significance level of 5%. No correction for multiple testing was performed due to the non-confirmatory design.

All endpoints were evaluated in the modified intention-to-treat (mITT) population, which includes all patients who underwent surgery because of their fracture in the group they were allocated to by randomization. Patients who underwent conservative treatment after randomization and therefore did not reach the primary endpoint were excluded from analysis. For the primary endpoint, additional sensitivity analyses were performed in the as-treated (AT) population. In the sensitivity analysis, patients were allocated to the intervention group or control group according to the protocol they were treated after mainly (AT), correcting for treatment changers from the intervention group to the control.

Multiple linear regression was performed to analyze a possible influence of the demographic variables (a) age, (b) gender, (c) education level, (d) comorbidities, (e) smoker, or (f) affected side on the primary endpoint.

Interval scaled variables were analyzed using Welch’s t-test. Ordinal scaled variables were analyzed using the Mann–Whitney U test with continuity correction. Fisher’s exact test was used in the case of categorical variables.

The evaluation was carried out using the program R in version 3.6.3.

The graphics were created in Prism program from GraphPad Software, version 8.3.1.

### Ethical approval

All procedures performed in this study involving human participants were in accordance with the ethical standards of the Ethics Committee of the State Medical Association of the Rhineland-Palatinate and with the 1964 Declaration of Helsinki and its later amendments or comparable ethical standards. This article does not contain any studies with animals performed by any of the authors.

### Informed consent

Informed consent was obtained from all individual participants included in the study. Additional informed consent was obtained from all individual participants for whom identifying information is included in this article. Data collection, coding, routing, and analysis were in accordance with legal data protection policy.

## Supplementary Information

Supplementary Information.

## Data Availability

The datasets generated and analyzed during the current study are available as a supplementary file 1.
